# Absolute quantitation of sympathetic nerve activity using [^123^I] metaiodobenzylguanidine SPECT-CT in neurology

**DOI:** 10.1186/s41824-024-00205-9

**Published:** 2024-06-01

**Authors:** Shintaro Saito, Kenichi Nakajima, Junji Komatsu, Takayuki Shibutani, Hiroshi Wakabayashi, Hiroshi Mori, Aki Takata, Kenjiro Ono, Seigo Kinuya

**Affiliations:** 1 Department of Nuclear Medicine, Kanazawa University, 13-1 Takara-machi, Kanazawa, 920-8640 Japan; 2 Department of Functional Imaging and Artificial Intelligence, Kanazawa University, Kanazawa, Japan; 3 Department of Neurology, Kanazawa University Graduate School of Medical Sciences, Kanazawa, Japan; 4 Department of Quantum Medical Technology, Institute of Medical, Pharmaceutical and Health Sciences, Kanazawa University, Kanazawa, Japan

**Keywords:** Heart-to-mediastinum ratio, Neurodegenerative disorder, Standardized uptake value, Three-dimensional quantitation, Washout rate

## Abstract

**Background and purpose:**

The ability of [^123^I]metaiodobenzylguanidine (MIBG) sympathetic nerve imaging with three-dimensional (3D) quantitation to clinically diagnose neurological disorders has not been evaluated. This study compared absolute heart counts calculated as mean standardized uptake values (SUV_mean_) using conventional planar imaging and assessed the contribution of [^123^I]MIBG single-photon emission computed tomography (SPECT)-CT to the diagnosis of neurological diseases.

**Methods:**

Seventy-two patients with neurological diseases were consecutively assessed using early and delayed [^123^I]MIBG SPECT-CT and planar imaging. Left ventricles were manually segmented in early and delayed SPECT-CT images, then the SUV_mean_ and washout rates (WRs) were calculated. Heart-to-mediastinum ratios (HMRs) and WRs on planar images were conventionally computed. We investigated correlations between planar HMRs and SPECT-CT SUV_means_ and between WRs obtained from planar and SPECT-CT images. The cutoff for SPECT-CT WRs defined by linear regression and that of normal planar WRs derived from a database were compared with neurological diagnoses of the patients. We assigned the patients to groups according to clinical diagnoses as controls (n = 6), multiple system atrophy (MSA, n = 7), progressive supranuclear palsy (PSP, n = 17), and Parkinson’s disease or dementia with Lewy bodies (PD/DLB, n = 19), then compared SPECT-CT and planar image parameters.

**Results:**

We found significant correlations between SPECT-CT SUV_mean_ and planar HMR on early and delayed images (R^2^ = 0.69 and 0.82, *p* < 0.0001) and between SPECT-CT and planar WRs (R^2^ = 0.79, *p* < 0.0001). A threshold of 31% for SPECT-CT WR based on linear regression resulted in agreement between planar and SPECT-CT WR in 67 (93.1%) of 72 patients. Compared with controls, early and delayed SUV_mean_ in patients with PSP and MSA tended more towards significance than planar HMR. This trend was similar for SPECT-CT WRs in patients with PSP.

**Conclusions:**

Absolute heart counts and SUV_mean_ determined using [^123^I]MIBG SPECT-CT correlated with findings of conventional planar images in patients with neurological diseases. Three-dimensional quantitation with [^123^I]MIBG SPECT-CT imaging might differentiate patients with PSP and MSA from controls.

## Background

The physiological noradrenaline analog, [^123^I]metaiodobenzylguanidine (MIBG), tracks absorption, transit, and the subsequent vesicular storage of noradrenaline in postsynaptic sympathetic nerve terminals. It can reveal sympathetic nerve activity, integrity, and disturbances in several pathological states. Myocardial [^123^I]MIBG uptake and washout are clinically useful markers of neurodegenerative disorders such as dementia with Lewy bodies (DLB) and Parkinson's disease (PD), as well as for evaluating disease severity and prognosis, the effects of treatment on heart failure, and arrhythmogenic disease (Nakajima and Nakata 2015; Nakajima and Yamada 2016; McKeith et al. 2017; Travin et al. 2017; Yamada et al. 2020; Verschure et al. 2022). Heart-to-mediastinum ratios (HMR) and washout rates (WR) determined from planar images are indicators of sympathetic nerve activity (Okuda et al. 2011; Nakajima et al. 2017; Owenius et al. 2017; Bateman et al. 2019). However, although assessment protocols have been standardized, the uncertainty of HMR analysis of clinical data results in up to 40% of patients remaining in the measurement gray zone (Klene et al. 2018). Three-dimensional (3D) images generated by single-photon emission computed tomography (SPECT) can distinguish between cardiac activity and background or overlapping organs. Quantitative 3D evaluation using [^123^I]MIBG SPECT is feasible without CT (Chen et al. 2012; Saito et al. 2021), and Lewy body disease has been diagnosed based on the heart-to-aorta ratio using [^123^I]MIBG SPECT-CT (Odagiri et al. 2016), which is a relative 3D quantitation method. When attenuation is corrected using low-dose CT, absolute heart counts and standardized uptake values (SUVs) in patients with cardiac and neurological diseases can be calculated based on [^123^I]MIBG SPECT images (Saito et al. 2023). However, the relationship between 3D quantitation and clinical diagnoses of neurological diseases remains unknown. Here, we aimed to determine the contribution of [^123^I]MIBG SPECT-CT to the differential diagnosis of neurological diseases. We therefore compared clinical data generated by conventional planar image-based quantitation with absolute heart counts determined as mean SUVs (SUV_mean_) derived from [^123^I]MIBG SPECT-CT images of patients with neurological diseases.

## Methods

### Patients

This study initially enrolled 72 consecutive patients (average age, 69.8 ± 8.8; range, 35‒88 years; male, n = 27; female, n = 45) with neurological diagnoses at Kanazawa University Hospital between 2020 and 2022. Table 1 shows that PS/PD/DLB (n = 50) comprised Parkinson’s syndrome (PS; n = 31), PD (n = 14), DLB (n = 5), and non-PS/PD/DLB (n = 22) including familial amyloid polyneuropathy (FAP; n = 7) and other neurological diseases. A neurologist with expertise in movement disorders categorized the diagnoses based on the recommended criteria for progressive supranuclear palsy (PSP), multiple system atrophy (MSA), PD, and DLB (Gilman et al. 2008; Postuma et al. 2015; Hoglinger et al. 2017; McKeith et al. 2017). Cardiac [^123^I]MIBG uptake was significantly reduced to a planar delayed HMR < 1.5 in 17 patients.

**Table 1:** Characteristics of 72 patients

Characteristics	n
Age	69.8 ± 8.8
Sex (male)	27 (38%)
Body weight (kg)	54.4 ± 11.5
Body mass index (kg/m^2^)	22.0 ± 3.5
PS/PD/DLB (n, %)	50 (69%)
Parkinson’s syndrome	31 (43%)
Progressive supranuclear palsy	17 (24%)
Multiple system atrophy	7 (9%)
Other Parkinson’s syndrome	7 (9%)
Parkinson’s disease	14 (19%)
Dementia with Lewy bodies	5 (7%)
Non-PS/PD/DLB	22 (31%)
Familial amyloid polyneuropathy	7 (9%)
Alzheimer disease	2 (3%)
Cerebral hemorrhage	1 (1%)
Other diseases and/or unknown etiology	12 (17%)
Reduced cardiac uptake (planar delayed HMR < 1.5)	11 (15%)

Data are shown as means ± SD or n (%)

*DLB* dementia with Lewy bodies, *HMR* heart-to-mediastinum ratio, *PD* Parkinson’s disease, *PS* Parkinson’s syndrome, *SD* standard deviation

### [^123^I]MIBG image processing

All patients were evaluated using early and delayed [^123^I]MIBG planar and SPECT-CT imaging. A medium-energy (ME) collimator was applied to image acquisition using Symbia Intevo and Symbia Intevo Bold SPECT-CT scanners (Siemens Healthcare, Erlangen, Germany). The patients were intravenously administered with 111 MBq of [^123^I] MIBG, and images were acquired 15–20 (early phase) and 180–240 (delayed phase) minutes later. Planar images were acquired for 5 min using 256 × 256-matrix with a zoom factor of 1.0 (2.4-mm pixels). The SPECT images were acquired using 128 × 128-matrix with a zoom factor of 1.0 (4.8-mm pixels), 60 projections, circular orbit of 360°, rotation radius of 24 cm, and 30 s per view. The SPECT data were reconstructed by an ordered subset conjugate gradient minimizer using the xSPECT Quant algorithm. Low-dose CT images were acquired for attenuation correction after SPECT image acquisition. The CT scanning parameters were: 130 kV, 20 mA with CARE Dose 4D, 16 × 1.2 mm collimation, 0.6-s rotation, and pitch, 1.5. Multiple-energy window scatter and CT-based attenuation correction was automated.

### SUVs and WRs in SPECT-CT images and left ventricular segmentation

**Fig. 1:**
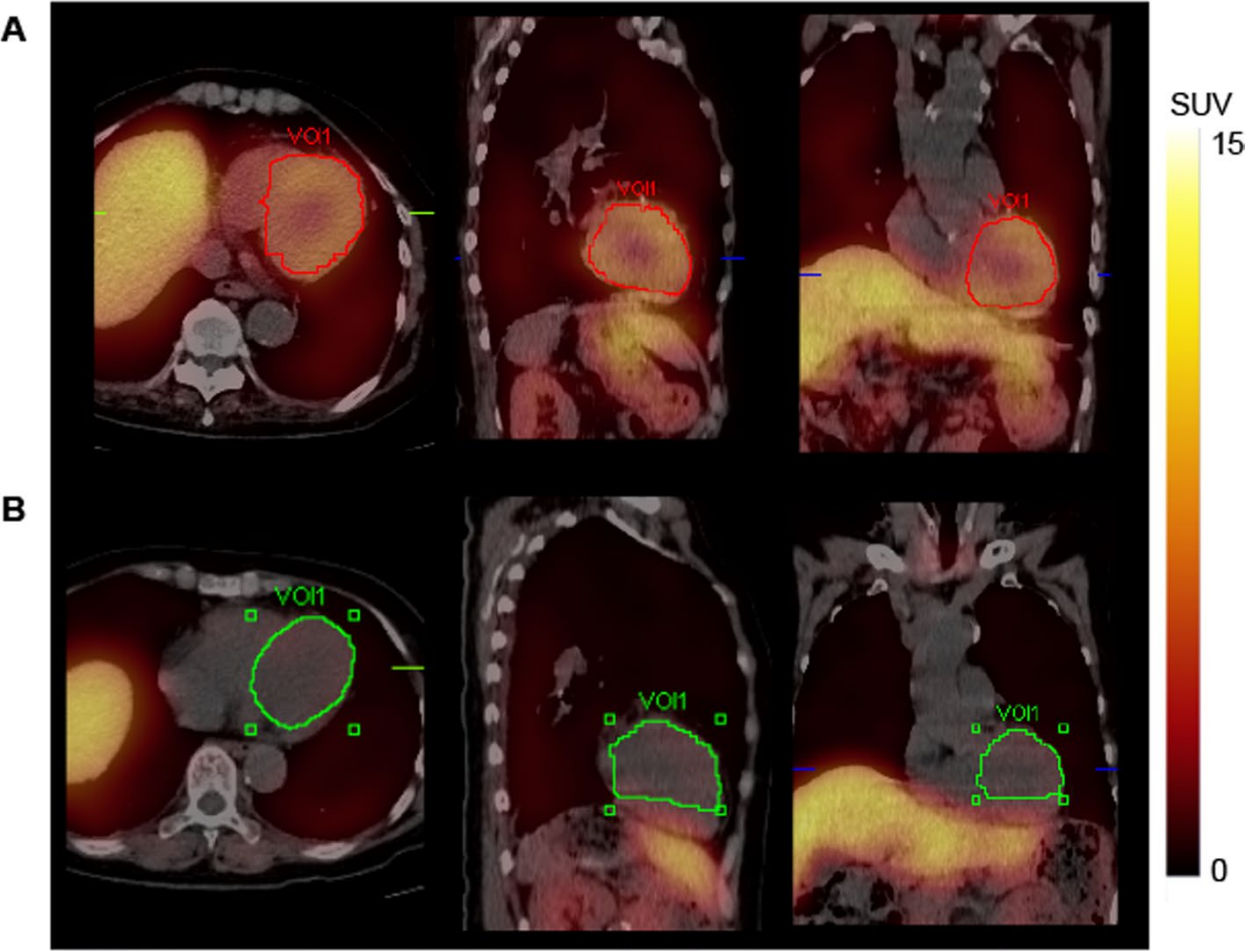
Uptake of [^123^I]MIBG and mean SUVs. Three-dimensional manual segmentation of left ventricle using [^123^I]MIBG SPECT-CT imaging. Normal **A** and reduced **B** [^123^I]MIBG uptake in 83- and 75-year-old female patients with cognitive dysfunction and autonomic neuropathy, respectively. Mean SUVs calculated from VOIs are 4.91 (**A**), and 1.01 (**B**). Red and green lines in VOI mark borders of manual 3D-segmented left ventricle. *3D* three-dimensional, *CT* computed tomography, *SPECT* single photon emission computed tomography, *SUV* standardized uptake value, *VOI* voxel of interest

We manually segmented entire left ventricle into regions of interest (ROI) in early and delayed SPECT-CT images as described (Saito et al. 2023). Two nuclear medicine specialists operated the SPECT and X-ray CT devices, and fusion imaging was applied to evaluate left ventricular morphology and [^123^I]MIBG activity, and to determine the boundaries of the left ventricle. Accumulation in the liver was clearly separated from the left ventricle for all segmentation processes. Figure 1 shows an example of 3D left ventricle segmentation. We calculated SPECT-CT SUV_mean_ using the SUV that reflected the normalized concentration of tissue radioactivity relative to the dose and weight of the injected radioactive tracer. This value represents the average SUV of voxels within volumes of interest (VOI) that included the complete left ventricle.

The SPECT-CT WRs were calculated based on SUV_mean_ values as:

$${\text{SPECT-CT WR}} = \left( {{\text{early SUV}}_{{{\text{mean}}}} - {\text{delayed SUV}}_{{{\text{mean}}}} } \right)/{\text{early SUV}}_{{{\text{mean}}}} \times {1}00 \, \left( \% \right).$$

### HMRs and WRs in planar images

Early and delayed HMRs were computed using semi-automatic SmartMIBG software (PDRadiopharma, Inc., Tokyo, Japan) for the planar images while ROIs were being established. (Okuda et al. 2011). The software algorithm uses a circular heart ROI and a mediastinal ROI with a 10% body width and a 30% mediastinum height. After the heart center is adjusted, the entire procedure is automated, with manual correction as required.

Planar WRs with time-decay corrections from early and delayed heart (H_early_ and H_delayed_) and mediastinal (M_early_ and M_delayed_) counts were calculated as:

$${\text{Planar WR}} = \, \left[ {\left( {{\text{H}}_{{{\text{early}}}} {-}{\text{ M}}_{{{\text{early}}}} } \right){-}\left( {{\text{H}}_{{{\text{delayed}}}} {-}{\text{M}}_{{{\text{delayed}}}} } \right)/0.5^{{({\text{t}}/13)}} } \right]/\left( {{\text{H}}_{{{\text{early}}}} {-}{\text{M}}_{{{\text{early}}}} } \right) \times {1}00\left( \% \right),$$

where t is the elapsed time (hours) between early and delayed image acquisition (Flotats et al. 2010).

### Quantitative analysis of SPECT-CT and conventional planar images

**Fig. 2:**
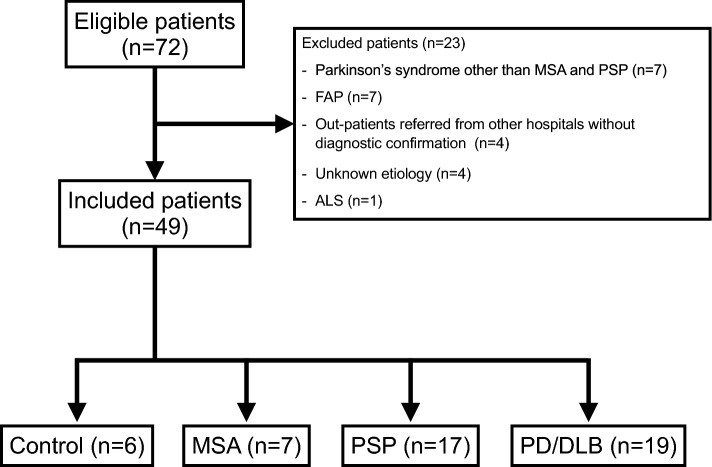
Flow-chart for classifying patients with neurological diseases into four groups. *ALS* Amyotrophic lateral sclerosis, *DLB* dementia with Lewy bodies, *FAP* familial amyloid polyneuropathy, *MSA* multiple system atrophy, *PD* Parkinson’s disease, *PSP* progressive supranuclear palsy

We analyzed correlations between SPECT-CT SUV_mean_ and planar HMRs in early and delayed images and between WRs derived from SPECT-CT SUV_mean_ and planar images. Based on standard values extracted from the JSNM working group databases (n = 62), the threshold for differentiating normal from abnormal planar WR was 34.0% (Nakajima et al. 2018). We determined a SPECT-CT WR cutoff by evaluating correlations between SPECT-CT and planar WRs, then used it to categorize patients as normal or abnormal. We subsequently assessed the degree of agreement between the methodologies. We assigned the participants (n = 49) to four groups based on clinical diagnoses of: Alzheimer's disease, depression, cerebral hemorrhage, cerebral palsy, and somatoform disorders (control; n = 6), MSA (n = 7), PSP (n = 17), and PD/DLB (n = 19). Figure 2 shows the flow of patient classification into these distinct groups. The exclusion criteria included: patients with FAP (n = 7), Parkinson’s syndrome other than MSA and PSP (n = 7), unknown etiology (n = 4), amyotrophic lateral sclerosis (n = 1) or outpatients referred from other hospitals without diagnostic confirmation (n = 4). The SUV_mean_ and WR calculated from SPECT-CT images and HMRs and WR calculated from planar images were compared among the four groups.

### Statistics

All data were analyzed using JMP Pro version 17 (SAS Institute Inc., Cary, NC, USA). Data are expressed as means and standard deviations (SD). Correlations between SUV_mean_ and HMR, and between SPECT-CT and planar WRs were assessed using t-tests and two-way analysis of variance. Values were compared between groups using t-tests. We assessed the diagnostic performance of MSA, PSP, and PD/DLB using SPECT-CT and planar images by the analysis of area under the receiver operating characteristics (ROC) curve (AUC). Values with *P* < 0.05 were considered significantly different.

## Results

### SPECT-CT SUV_mean_ versus planar HMR and SPECT-CT WR versus planar WR

**Fig. 3:**
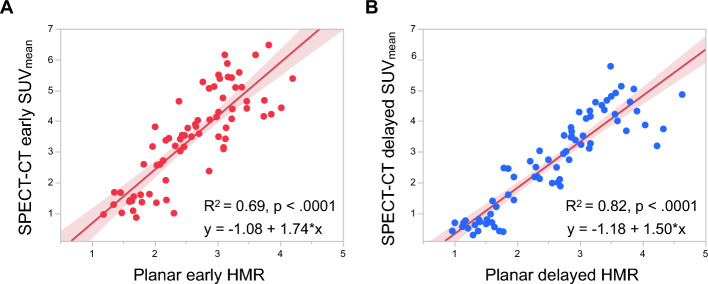
Correlations between SUV_mean_ and HMR. Correlations between SUV_mean_ derived from [^123^I]MIBG SPECT-CT images using manual 3D left ventricle segmentation and HMR from planar images using conventional methods. Early SPECT-CT-derived SUV_mean_ versus planar early HMR (**A**); Delayed SPECT-CT-derived SUV_mean_ versus planar delayed HMR (**B**). Shaded area, confidence of fit. *3D* three-dimensional, *CT* computed tomography, *HMR* heart-to-mediastinum, *SPECT* single photon emission computed tomography, *SUV* standardized uptake value

**Fig. 4:**
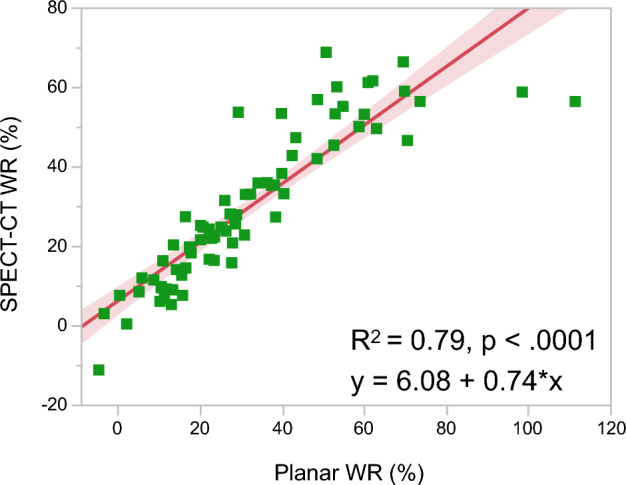
Correlations between WRs derived from [^123^I]MIBG SPECT-CT images and planar images. SPECT-CT WR using SUV_mean_ versus planar WR. Shaded area, confidence of fit. SPECT, single photon emission computed tomography, SUV, standardized uptake value; WR, washout rate

The SUV_mean_ was calculated from early and delayed [^123^I]MIBG SPECT-CT images acquired from all the patients. Figure 1 shows examples of manual 3D left ventricular segmentation and the resulting SUV_mean_. We compared SPECT-CT SUV_mean_ and planar HMR on early and delayed [^123^I]MIBG images. The early and delayed SPECT-CT SUV_mean_ significantly correlated with planar HMR (R^2^ = 0.69 and 0.82, *p* < 0.0001; Fig. 3). We also compared SPECT-CT WRs using SUV_mean_ and planar WR using conventional methods. significantly correlated between planar and SPECT-CT WRs (R^2^ = 0.79, *p* < 0.0001; Fig. 4). We determined a 31% threshold for SPECT-CT WRs using planar WR linear regression with a maximum standard deviation of 34% (Nakajima et al. 2018). Normal and abnormal WRs that were subsequently classified based on the cutoff values for SPECT-CT and planar WRs agreed in 67 (93.1%) of 72 patients (Table 2).

**Table 2:** Washout rates derived from SPECT-CT images using 3D manual segmentation and planar images using conventional method

		Planar WR		Total
		< 34%	≥ 34%	
SPECT-CT WR	< 31%	40 (55.5%)	1 (1.4%)	41 (56.9%)
	≥ 31%	4 (5.6%)	27 (37.5%)	31 (43.1%)
Total		44 (61.1%)	28 (38.9%)	72

SPECT-CT washout rates calculated from early and delayed SUV_mean_

*3D* three-dimensional, *CT* computed tomography, *SPECT* single photon emission computed tomography, *WR* washout rate

### Comparison of SPECT-CT and planar image parameter values between groups

Table 3 shows comparisons of [^123^I]MIBG SPECT-CT and planar parameters between groups. Early and delayed SUV_mean_ and WR in SPECT-CT images significantly differed between patients with PD/DLB and controls (*p* = 0.0006, *p* < 0.0001, and *p* = 0.0003, respectively). The statistical significance of early and delayed HMR and WR in planar images between patients with PD/DLB and controls was similar to that of the SPECT-CT parameters (*p* = 0.0153, *p* = 0.0021, and *p* = 0.0011, respectively). Early and delayed SUV_mean_ and WRs tended to differ more than planar parameters in patients with PSP compared with controls (*p* = 0.195, *p* = 0.0845, and *p* = 0.0644, respectively). The trends of early and delayed SUV_means_ (*p* = 0.125 and *p* = 0.132, respectively) were similar between patients with MSA and controls. The SPECT-CT parameters significantly differed between patients with PSP or MSA and those with PD/DLB, except for early SUV_mean_ between patients with MSA and PD/DLB. Early and delayed HMRs and WRs significantly differed between planar images of patients with PSP or MSA and those with PD/DLB. No significant differences were found between PSP and MSA. Diagnoses of MSA, PSP, and PD/DLB, ROC derived using planar and SPECT-CT parameters did not significantly differ.

**Table 3:** Comparisons groups using [^123^I]MIBG SPECT-CT and planar parameters

A. Mean ± SD of parameters				
	PD/DLB	PSP	MSA	Control
	(n = 19)	(n = 17)	(n = 7)	(n = 6)
Early SUV_mean_	2.40 ± 1.19	3.83 ± 1.52	3.52 ± 1.21	4.65 ± 1.00
	0.86‒5.09	1.00‒5.58	1.01‒4.43	3.19‒6.15
Delayed SUV_mean_	1.50 ± 1.02	2.96 ± 1.52	2.94 ± 1.21	3.99 ± 0.81
	0.29‒3.79	0.40‒5.58	0.51‒3.87	2.69 ‒4.91
SPECT‒CT WR (%)	43.3 ± 15.4	28.3 ± 18.2	20.0 ± 16.4	14.0 ± 4.34
	20.7‒66.3	5.2‒68.7	2.9‒49.5	7.5‒20.2
Early HMR	2.13 ± 0.61	2.81 ± 0.58	2.90 ± 0.89	2.88 ± 0.47
	1.19‒3.29	1.61‒3.86	1.48‒4.02	2.33‒3.61
Delayed HMR	1.88 ± 0.60	2.78 ± 0.85	3.04 ± 1.07	3.04 ± 0.47
	0.97 ‒2.86	1.18‒4.33	1.22 ‒4.23	2.21‒3.57
Planar WR (%)	46.2 ± 21.9	28.4 ± 17.7	22.2 ± 21.6	14.5 ± 10.3
	16.5‒111.5	8.8‒70.6	‒3.3‒63.1	0.5 ‒27.8

B. Results of t tests (*p* values)						
	PD/DLB versus Control	PSP versus Control	MSA versus Control	PD/DLB versus PSP	PD/DLB versus MSA	MSA versus PSP
Early SUV_mean_	0.0006	0.195	0.125	0.0019	0.0587	0.588
Delayed SUV_mean_	< 0. 0001	0.0845	0.132	0.0009	0.0109	0.971
SPECT-CT WR (%)	0.0003	0.0644	0.503	0.0068	0.0018	0.249
Early HMR	0.0153	0.819	0.941	0.0025	0.0082	0.738
Delayed HMR	0.0021	0.485	0.998	0.0008	0.0012	0.462
Planar WR (%)	0.0011	0.138	0.482	0.009	0.0076	0.476

C. Area under the ROC curve (AUC) and *p* values obtained from ROC analysis						
	Early SUV_mean_	Early HMR	Delayed SUV_mean_	Delayed HMR	SPECT-CT WR	Planar WR
MSA	0.548	0.654	0.590	0.691	0.704	0.682
p	0.205		0.128		0.560	
PSP	0.654	0.660	0.631	0.643	0.570	0.580
p	0.914		0.792		0.781	
PD/DLB	0.804	0.790	0.822	0.840	0.821	0.809
p	0.796		0.624		0.710	

Data are shown as means ± standard deviations with ranges

*AUC* area under the ROC curve, *CT* computed tomography, *DLB* dementia with Lewy bodies, *HMR* heart-to-mediastinum ratio, *MSA* multiple system atrophy, *PD* Parkinson’s disease, *PSP* progressive supranuclear palsy, *ROC* receiver operating characteristic, *SD* standard deviation, *SPECT* single photon emission computed tomography, *SUV*_*mean*_ mean standardized uptake value, *WR* washout rate

## Discussion

The present findings revealed a close correlation between planar imaging-based quantitative and SUV_mean_ values obtained from [^123^I]MIBG SPECT-CT images of patients with neurological diseases. We confirmed that [^123^I]MIBG SPECT-CT revealed consistently reduced uptake in patients with PD/DLB in general. Quantitative evaluation using [^123^I]MIBG SPECT-CT also helped to differentiate MSA and PSP from controls.

### Absolute quantitation using SPECT-CT

Absolute quantitation using 3D images could be one option for analyzing cardiac sympathetic images. Standard cardiac sympathetic imaging is not completely quantitative and is assessed by comparing the heart to other tissues. Semi-quantitative standard visual scoring systems that compare the heart to the mediastinum improves visual scoring but relies on the extracardiac position as a reference area. In addition, anatomically overlapping organs on two-dimensional (2D) images are avoided on 3D images. Calculating the absolute amount of myocardial uptake using quantitative SPECT might further improve diagnostic performance by narrowing the gray zone in conventional 2D quantitative evaluation. Nuclear medicine quantitation has progressed from simple thyroid uptake measurements to PET-based kinetic and SUV analyses. The need for radionuclide therapy has primarily led to SPECT quantitation becoming more prevalent. The potential applications of absolute SPECT-CT image quantitation such as myocardial perfusion, ^99m^Tc-Pyrophosphate scintigraphy, and cardiac sympathetic nerve imaging to diagnoses have been discussed (Ramsay and Cuscaden 2020; Dorbala et al. 2021; Lehner et al. 2022; Nakajima et al. 2023; Saito et al. 2023). These applications should become more established in the field of nuclear medicine diagnostics.

### Application of SUV_mean_

Activity normalization within an ROI is represented by SUVs, which comprise the amount of injected activity divided by that distributed within a patient. The significant advantage of quantitative evaluation using SUVs is that the calculated values are independent of body weight. Standard uptake values vary. Here, we calculated SPECT-CT SUV_mean_ as the average SUV of voxels inside a VOI that comprised the entire left ventricle (Saito et al. 2023). The following might offer a possible explanation. The maximum standardized uptake value (SUV_max_) can serve as a quantitative measure when assessing [^123^I]MIBG sympathetic images. However, SUV_max_ is derived from a single pixel rather than an entire ROI. When SUV_max_ is calculated for [^123^I]MIBG sympathetic images of the heart, this could result in significant deviation. The myocardial uptake of [^123^I]MIBG is variable and can be affected by inferior deficiencies or diffusely decreased uptake with Lewy body disorders. Another reason for using SUV_mean_ is that the connection is closer to SUV_mean_ than SUV_max_ compared with 2D quantitative values in patients with neurological and cardiac disorders (Saito et al. 2023). We identified a close correlation between SUV_mean_ and planar HMR in patients with neurological diseases. The correlation was also good for SUV_mean_ and WRs. The SUV_mean_ in [^123^I]MIBG sympathetic imaging could be a valuable quantitative indicator of myocardial accumulation. However, the average SUV depends on the definition of the ROI and might be affected by intra- and inter-observer variability.

### Differentiation of PSP, MSA, and controls on cardiac sympathetic nerve images

We determined that quantifying sympathetic neural function in 3D SPECT-CT images might be useful to differentiate patients with PSP and MSA from controls. Although 3D evaluation found no significant differences, the SUV_mean_ tended to be lower and the WR tended to be higher in patients with PSP than controls, and was stronger than the conventional 2D findings (*p* = 0.195, *p* = 0.0845 and *p* = 0.0644, respectively; Table 3). A trend towards lower early and delayed SUV_mean_ values in patients with MSA *versus* controls was also more obvious in 3D, than conventional 2D images (*p* = 0.125 and *p* = 0.132, respectively; Table 3). Planar images of patients with PSP have revealed slightly reduced MIBG accumulation, which might be associated with brainstem atrophy (Kamada et al. 2019). Although cardiac [^123^I]MIBG uptake is thought to be primarily maintained in MSA with prejunctional autonomic failure, the planar HMR was decreased compared with controls during the early and delayed phases in 5 and 4 of 9 patients with Parkinson's variant of multiple system atrophy (MSA-P) (Kollensperger et al. 2007). Reduced [^123^I]MIBG accumulation in MSA might be partly explained by factors associated with Lewy body pathology (Nagayama et al. 2010). The elimination of organ overlaps and mediastinal dependence in 3D quantitative assessment might have resulted in a more precise reflection of sympathetic nerve function. Because the SUV_mean_ is based on absolute quantitation and not ratios as in HMR, it has a wider range than conventional HMR values and could be subdivided into normal, PSP, MSA, and PD/DLB. Currently, HMR derived from planar images is the most popular measure of sympathetic nerve activity, whereas SPECT data are considered supplementary. However, a diagnostic system that includes SPECT might establish 3D quantitative assessment as the diagnostic standard. Moreover, data collection by tomographic imaging is a standard procedure for semiconductor SPECT systems that do not rely on traditional planar images. Consequently, direct use of SPECT images could be a practical alternative.

### The future

We manually segmented the heart including the entire left ventricular cavity and the myocardium because our objective was to determine the contribution of quantified 3D values to the diagnosis of neurological diseases. We therefore compared conventional 2D, with 3D quantitative values using SPECT-CT. Isolating the myocardium using non-contrast X-ray CT is challenging. The entire left ventricle was segmented because preserved and decreased accumulation must be considered when [^123^I]MIBG is used to assess sympathetic nerve function. Subsequent investigations will require finding a way to separate and isolate the myocardium from the left ventricular cavity. Since manual segmentation is complicated and requires prolonged processing, integrating artificial intelligence with automatic 3D segmentation should significantly enhance convenience. Cardiac segmentation and quantitation by [^123^I]MIBG SPECT has been based on a convolutional neural network (Saito et al. 2021). This technique should be extended to [^123^I]MIBG SPECT-CT imaging using segmentation and quantitation automated by artificial intelligence.

### Limitations

The possibility of unintentional selection bias could not be entirely excluded due to our small patient cohort and the retrospective, single-center study design. The current investigation is an initial attempt to assess whether quantitative 3D assessment of [^123^I] MIBG sympathetic images could differentiate neurological diseases based solely on their diagnoses. This study included consecutive cases with diverse backgrounds, and did not focus on a specific disorder. Our expert neurologist retrospectively determined the most likely neurological diagnoses based on clinical evaluations during hospital stays and outpatient visits. While this approach of consecutive inclusion might introduce some level of diagnostic uncertainty, it reflects real-world clinical practice. Prospective, large-scale investigations are needed to validate the diagnostic accuracy of [^123^I]MIBG scintigraphy using SPECT/CT to account for disease progression and severity.

## Conclusions

Absolute cardiac counts and SUVs significantly correlated between [^123^I]MIBG SPECT-CT and conventional quantitative planar images of neurological diseases. This outcome indicated that a differential diagnosis between controls and patients with PSP and MSA would require 3D quantitative evaluation using [^123^I]MIBG SPECT-CT. Absolute 3D quantitation of sympathetic innervation is a viable approach that could be integrated into methods to quantify cardiac sympathetic activity.

## Data Availability

Without authorization from the Ethics Committees of Kanazawa University, the image datasets generated or analyzed in this research are not disclosed publicly. However, the corresponding author may provide them upon reasonable request.

## Abbreviations

AUC: Area under the ROC curve
DLB: Dementia with Lewy bodies
HMR: Heart-to-mediastinum ratio
MIBG: Metaiodobenzylguanidine
MSA: Multiple system atrophy
PD: Parkinson’s disease
PS: Parkinson’s syndrome
SPECT: Single-photon emission computed tomography
ROC: Receiver operating characteristic
SUV: Standardized uptake value
WR: Washout rates
